# Spontaneous Remission of Congenital Complete Atrioventricular Block in Anti-Ro/La Antibody-Negative Monozygotic Twins: Case Report

**DOI:** 10.4274/balkanmedj.2015.0535

**Published:** 2017-01-05

**Authors:** Taner Kasar, Murat Saygı, İsa Özyılmaz, Yakup Ergül

**Affiliations:** 1 Department of Pediatric Cardiology, İstanbul Mehmet Akif Ersoy Thoracic and Cardiovascular Surgery Center and Research Hospital, İstanbul, Turkey

**Keywords:** Anti-Ro/La antibody, congenital heart block, discordant complete atrioventricular block, infants

## Abstract

**Background:**

Congenital complete atrioventricular block without any structural heart disease and anti-Ro/La negativity is very rare. Discordant complete atrioventricular block, which is more frequently defined in the literature as an autoimmune mechanism, is much more rare in monozygotic twins.

**Case Report:**

The 26-year-old healthy mother had given birth in her first spontaneous, uneventful pregnancy to monozygotic twins at week 35. While the first twin’s physical examination proved her to be normal with a pulse rate consistent with her age, the second twin had a pulse rate of approximately 40 beats/minute.The patient was confirmed to have congenital complete atrioventricular block.

**Conclusion:**

Despite this case appears to be an isolated one, a discordant complete atrioventricular block regression without any autoimmune evidence should be included in the differential diagnosis of bradycardia in infants.

Congenital complete atrioventricular block (CAVB) is seen in approximately one in every 20.000 live births. Congenital CAVB is rarer in patients that are anti-Ro/La negative or who do not exhibit any structural heart disease. More than 90% of congenital atrioventricular blocks (AVB) are accompanied by maternal autoimmune antibodies or structural heart disease. The remaining 10% are idiopathic AVB (1). Congenital CAVB in monozygotic twins is rarer, and usually defined in the literature as an autoimmune mechanism (2,3). We present here a case of discordant CAVB that sustained remission in monozygotic twin infants who were autoimmune negative and did not have any structural heart disease. To the best of our knowledge, this is the first to report discordant CAVB regression as shown in monozygotic twins with no autoimmune evidence.

## CASE PRESENTATION

**Figure 1:**
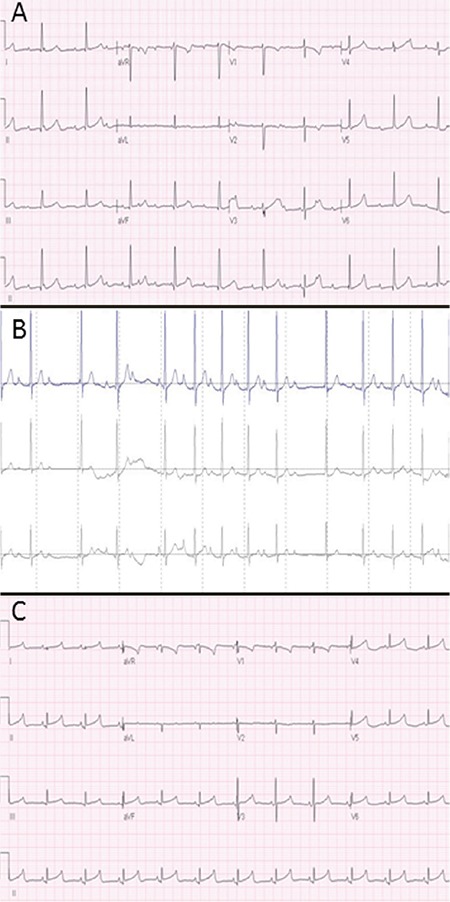
a, b, c. Day 1: Complete AV dissociation; atrial rate of 166/minute, ventricular rate of 61/minute, QRS duration of 0.084 seconds (occasionally >0.08 seconds), corrected QT interval of 0.43 seconds (a). Day 15: 24-hour Holter recording Wenckebach type II AV block (b). Sixteen month follow-up visit: Normal sinus rhythm (c).

The 26-year-old healthy mother had given birth in her first spontaneous, uneventful pregnancy to monozygotic twins at week 35, one of whom had a birth weight of 2.320 grams. The mother did not have a history of infection, metabolic disease, autoimmune disease, or drug usage during the pregnancy period. After the evaluation of the fetuses at 35th week of gestation, an emergency cesarean section was performed on the diagnosis of fetal distress due to bradycardia of the index fetus which was determined by a non-stress test. Following birth, both babies had normal Apgar scores. While the first twin’s physical examination proved her to be normal with a pulse rate consistent with her age, the second twin had a pulse rate of approximately 40 beats/minute; therefore, the twin with bradycardia was hospitalized. Informed consent was obtained from the parents at this time. The patient was confirmed to have CAVB by 12-lead electrocardiography (ECG) (MAC 1600, GE Healthcare, USA) and 24-hour Holter monitorization (Life card CF, Del Mar Reynolds Medical, United Kingdom) (Figure 1a). No structural cardiac defect was observed on the echocardiogram (ECHO) (Philips IE33; USA). Serum electrolyte levels, cardiac enzymes, and pro-brain natriuretic peptide levels were normal, and there were no findings of congestive heart failure (shortening fraction: 36%). Viral serology markers for myocarditis etiology were negative. Isoproterenol (HOSPIRA, INC., Lake Forest, USA) infusion was initiated for significant bradycardia (35-40/minute). Following treatment with isoproterenol, a normal sinus rhythm with a heart rate of 120/minute was reached; therefore, treatments were discontinued. Two days later, the heart rate dropped back to 50 beats/minute and was confirmed by a 12-lead ECG. Isoproterenol treatment was restarted and then discontinued one week later as the majority of the rhythm was sinus. On the fifteenth day of the follow-up, the patient was confirmed to have a normal sinus rhythm with rare Wenckebach type II AVB (Figure 1b). As the patient’s overall condition and vital signs were stable with normal cardiac functions, the patient was discharged. The ECG and ECHO findings of the mother and the other twin were normal.

The immunological markers, including anti-Ro/SSA, anti-LA/SSB, anti-DNA, antinuclear antibody, anti-cardiolipin antibody, anti-Sm, U1-RNP, Jo-1 and Scl70, of both infants and the mother were found to be negative. Anti-Ro/SSA and anti-LA/SSB immunoblotting yielded negative results in a reference laboratory with ISO 15189 accreditation.

At the first and sixth month follow-up visits, the auto-antibodies of the patient and the mother were checked for late seroconversion and were found to be negative. At the sixth month follow-up visit, both Holter monitoring and the ECG (Figure 1c) indicated a normal sinus rhythm.

## DISCUSSION

Congenital CAVB is rare in patients who are anti-Ro/La negative and have no concomitant structural heart diseases (3). Brucato et al. (4) showed that 20% of unselected congenital AVB have anti Ro/La-negative mothers, and Maeno et al. (5) identified this rate at 18%. In these studies, two fetuses were reported to have second degree AVB, with one diagnosed immediately after birth, and progressive AVB was demonstrated at the third month follow-up in the other three fetuses. Two other infants were noted as having alternating block with normal sinus rhythm; in one infant, stable, normal sinus rhythm was reportedly restored. Breur et al. (6) reported a series of four patients with fetal heart block, structural heart defects, and negative maternal antibodies (anti-Ro or anti-La antibodies). The neonates were reported to have an unstable progress of the AVB pattern with an occasional block of the sinus rhythm at varying degrees. Our patient had CAVB at birth and varying degrees of AVB, including the sinus rhythm during follow-up, which was detected in Holter monitorization. At the sixth month follow-up visit, normal sinus rhythm was observed in the ECG and the 24-hour Holter monitor recording.

Previous studies have reported that negative immune complex (anti-Ro or anti-La antibodies) findings do not always rule out an immune-mediated event; anti-Ro and anti-La antibodies can exhibit a stable profile for many years, but late seroconversion may remain a risk (7). Methods that are not sensitive and low concentrations of maternal antibodies may be numbered among potentially significant factors in this respect. The reason for late seroconversion is considered to be influenced by anti-Ro and anti-La antibodies as well as by unknown intrinsic (fetal) or extrinsic (maternal) factors (3). In our case, the levels of antibodies in the mother and infants, which were measured immediately after birth and at months one and six for the possibility of late seroconversion, were all found to be negative. On the other hand, other studies specify that maternal antibodies may not constitute an adequate reason for AVB, and it was shown that infants of seropositive mothers had an incidence rate of approximately 1-7.5% (8).

Discordant congenital CAVB is a rare occurrence in monozygotic twin infants (2). To the best of our knowledge, there is no published report of congenital CAVB in autoimmune negative twins. The reason for this discordance in twins remains unknown. However, the fact that autoimmune positive mothers reported to have children with congenital block only at a rate of 1-7.5% shows that the autoimmune mechanism would fall short of accounting for this situation. This result suggests that the discordant status may be related to unknown intrinsic (fetal), extrinsic (maternal), and/or environmental factors.

Furthermore, there are conflicting reports about the prognosis of congenital AVB patients. According to Berg et al. (8), the mortality rate was found to be similar among children with congenital AVB who were anti-Ro negative and children with congenital AVB who were anti-Ro positive. A series of studies exists which demonstrate the spontaneous improvement of congenital heart block without any cardiomyopathy within the follow-up period (6). In the light of this information, it is safe to say that congenital AVB patients may not always require urgent pacemaker implantation. Citing the most recent guidelines, symptomatic bradycardia with congenital AVB has been accepted as a Class I indication for pacemaker implantation in cases such as wide QRS escape and complex ventricular ectopy (9). Another Class I pacemaker indication in this group is basal heart rate: a ventricular rate below 55 beats/minute for an infant or a ventricular rate below 70 beats/minute in an infant with congenital heart disease. Although the original studies reporting these thresholds did not have 24-hour Holter data, this is frequently interpreted as an average 24-hour heart rate (10). We chose to include a clinical follow-up for our patient because the average heart rate was high when evaluated by 24-hour Holter ECG monitoring. Even though the initial heart beats were around 40/minute, the patient had no structural heart disease, asymptomatic and cardiac functions were fine, on ECHO evaluation. Pacemaker implantation is not a risk-free procedure, especially in infants as pacemaker implantation requires open heart surgery, the pacemaker generator needs space, and has risk of infection. We preferred to monitor our asymptomatic patient with more frequent follow-up visits and 24-hour Holter monitorization.

Our conclusion is that discordant congenital CAVB may develop in monozygotic twins born to an autoimmune negative mother. The pathogenesis of this condition is still unknown. If the cardiac functions of such patients are normal, they may be clinically monitored until normal sinus rhythm is restored.
